# The implications of evolutionary changes in the dendritic morphology of cerebellar Purkinje cells for information processing

**DOI:** 10.1186/1471-2202-14-S1-P373

**Published:** 2013-07-08

**Authors:** Kirsty Kidd, Hugo Cornelis, James M Bower, Daniel Polani, Neil Davey, Volker Steuber

**Affiliations:** 1 School of Computer Science, University of Hertfordshire, Hatfield, Hertfordshire, AL10 9AB, UK; 2 University of Texas Health Science Center, San Antonio, Texas, 78245, USA; 3 Barshop Institute for Longevity and Aging Studies, San Antonio, Texas, 78245, USA

## 

The dendritic morphology of neurons has been a focus of study in computational neuroscience for many years (e.g. [1]), but it is rare that the morphology of the same cell has been compared across different species. This study compares the morphology and resulting predicted passive electrical properties of cerebellar Purkinje cells obtained from a variety of different animal species, both mammalian and non-mammalian. The comparison is based on single cell reconstructions incorporated into the GENESIS simulation platform. For comparison purposes, all simulations were performed using values for membrane conductance and axial resistivity applied in previous models of mammalian Purkinje cells [2]. In addition to quantifying differences in morphology and expected passive electrical properties, the possible information processing capability of each species of Purkinje cell was evaluated using information theoretical techniques such as those used previously to quantify relationships between input and output [3]. The resulting information theoretical measures are then used to compare the performance of each cell, and to identify compensatory mechanism that could preserve the information processing capabilities in the presence of the morphological changes that occur during evolution.
